# Impacts of Regional Speed Control Strategy Based on Macroscopic Fundamental Diagram on Energy Consumption and Traffic Emissions: A Case Study of Beijing

**DOI:** 10.3389/fpubh.2022.883359

**Published:** 2022-06-24

**Authors:** Wensi Wang, Zirui Wang, Guangjun Wang, Bin Yu, Yuhe Xu, Kun Yu

**Affiliations:** ^1^Beijing Advanced Innovation Center for Big Data and Brain Computing, Beihang University, Beijing, China; ^2^CIECC Overseas Consulting Co., Ltd., Beijing, China; ^3^China Railway Construction Investment Group Co., Ltd., Beijing, China; ^4^Chongqing Communications Planning Surverying & Designing Institute, Chongqin, China

**Keywords:** urban built roads, PM2.5 exposures, energy consumption, Macroscopic Fundamental Diagram, respiratory health

## Abstract

Numerous studies shown that particulate matter in the ambient environment has a significant impact on the health of the respiratory system. To understand the interrelationships between urban built environment, transportation operations and health, this study proposes an innovative approach that uses real-world GPS datasets to calculate energy consumption and emissions from transportation. The experiment used the traffic operation state in the Fourth Ring Road of Beijing as the research object and tested the impact of using the Regional speed optimization (RSO) strategy based on Macroscopic Fundamental Diagram (MFD) on energy consumption and emissions during peak hours. The impact of traffic emission on the health of roadside pedestrians is also considered. Changes in PM2.5 concentrations around four different built-up areas were calculated and compared. The computational experiments indicate that the PM2.5 pollutants exhausted by the traffic on the Ring Road during peak hours can reach up to 250 μ*g*/*m*^3^, while the traffic emission on general roads near residential areas is only 50 μ*g*/*m*^3^. Adopting Regional speed optimization can reduce the energy consumption of the road network by up to 18.8%. For roadside runners, the PM2.5 inhalation caused by night running in commercial and recreational areas is about 1.3-2.6 times that of night running in residential areas. Compared with morning or night running, the risk of respiratory disease caused by PM2.5 inhalation was about 10.3% higher than commuter running behavior. The research results provide a useful reference for energy conservation and emission reduction control strategies for different road types in cities and help existing cities to establish a traveler health evaluation system caused by traffic operation.

## Introduction

In the world energy consumption structure, the transportation industry is always an important role (1). The transport sector accounted for 40% of Portugal's final energy consumption in 2013, with the road transport sector accounting for 81% of this energy consumption (2). This is mainly due to the dependence of transportation on fossil fuels. Meanwhile, various types of particulate matter produced during the combustion of fossil fuels are also one of the important sources of air pollution and become one of the hotspots that people pay attention to (3–5). Particulate matter (PM) pollution such as PM10 and PM2.5 have been shown to reduce respiratory function and other adverse health effects, leading to increased mortality (6–10), especially among the infirm and the elderly (11). The study by Chen et al. (12) showed that exposure to environmental PM1, PM2.5 and PM10 pollution had severe adverse effects on hospitalization for ischemic stroke, 3.5, 3.6, and 4.1% of hospital admissions for ischemic stroke could be attributable to PM1, PM2.5 and PM10, respectively. Yang et al. investigated the association between PM1 and PM2.5 and CVD prevalence in Chinese adults by randomly recruiting 24,845 adults aged 18-74 years from 33 communities in Northeast China. The results show that long-term PM1 exposure was positively related to CVD, especially in men and the elder (13). Lu et al. systematically searched the PubMed, Web of Science, and China National Knowledge Infrastructure databases using as keywords names of 127 major cities in Mainland China, Hong Kong, and Taiwan and finally screened 59 articles. Random-effects, fixed-effects models and Meta regression were run to explore the association between exposure to particulate matter with aerodynamic diameters <10 and 2.5 mm and health effects. The result shows that a 10 μ*g*/*m*^3^ increase in PM2.5 was associated with a 0.40% (95%CI: 0.22%, 0.59%) increase in total non-accidental mortality, a 0.63% (95%CI: 0.35%, 0.91%) increase in mortality due to cardiovascular disease, and a 0.75% (95%CI: 01.39%, 1.11%) increase in mortality due to respiratory disease (14). Health concerns drive growing awareness of the importance of controlling urban transport energy consumption and emissions (15–17). The study by (18) confirms that in Madrid traffic emissions have a major impact on PM2.5 concentrations in the urban environment. Similar conclusions have also been proved to exist in Toronto by (19). The impact of traffic PM2.5 emissions on human health cannot be ignored. The deposition method of traffic particles is diffusion sedimentation (20). After entering the human respiratory tract fine traffic particles deposited in the deep or pulmonary region of the lungs. The study also shows that people who are located near the road are more susceptible to the effects of fine particulate matter from traffic. Ling et al. proposed a hierarchical analysis framework, which analyzes several problems of different traffic related behaviors from top to bottom, including the purchasing behavior of new energy vehicles, the selection behavior of green travel modes and the behavioral reactions to TDM policies by dividing the management of transportation system into four levels from macro to micro. A useful management insight for traffic managers was provided to guide the design and improvement of sustainable traffic management policies (21). PM pollution in cities also affected by the built environment such as the diversity of urban area land use, street connectivity, building density and accessibility (22–24). Therefore, green buildings that can reduce emissions have also become one of the research hotspots (25). Some previous studies have focused on how driving patterns affect emissions and fuel consumption (26, 27). For example, Brundell-Freij and Ericsson (28) used a dataset of actual traffic driving patterns to assess the impact of parameters such as street characteristics, vehicle performance, driver category on driving patterns as well as emissions and fuel consumption.

Traffic operation have a significant impact on fuel consumption and emissions. Fuel consumption increases in the presence of traffic congestion, which has been found in previous studies (29–31). However, the research on the large-scale urban road network is still insufficient.

Since the traffic operation is always in dynamic change, even on the same road, there are obvious differences in the actual traffic characteristics on the road caused by the traffic demand at different time periods. Therefore, when measuring the emission generated by urban traffic, the dynamic change of road network operation state is a factor that must be considered. In traditional emission estimation of urban road network level, most of them estimate the total energy consumption and emission of the road network through the total number of vehicles. This static calculation method can only not effectively reflect the emission and energy consumption characteristics of traffic between different roads or at traffic nodes in the road network.

To expand the existing research to the road network level and enrich the research content in this field, this study uses vehicle GPS data and built road data to estimate PM2.5 and energy consumption from traffic. Taking the road section as the basic unit for calculating emissions and considering the working state of the engine under factors such as traffic congestion to calculate the emission rate of the road section at the average speed of a certain vehicle. Compared with previous studies (32, 33), this study further refined the PM2.5 impact caused by traffic by considering different urban built-up road traffic conditions, which is helpful to analyze the emission impact and energy consumption of different roads in the urban built-up environment. The main contributions of this study are as follows:

(a) A network-level traffic emission and energy consumption model with traffic operation state as input is established. PM2.5 emissions from traffic operations in urban built environment can be calculated.

(b) According to the characteristics of traffic operation in the city, a Regional speed optimization is proposed based on the above model. This strategy can effectively reduce vehicle energy consumption and PM2.5 emissions. At the same time, the health effects of emissions from traffic in different regions on roadside exercisers (such as joggers) were further analyzed.

## Methodology and Data Collection

### Methodology

**Figure 1:**
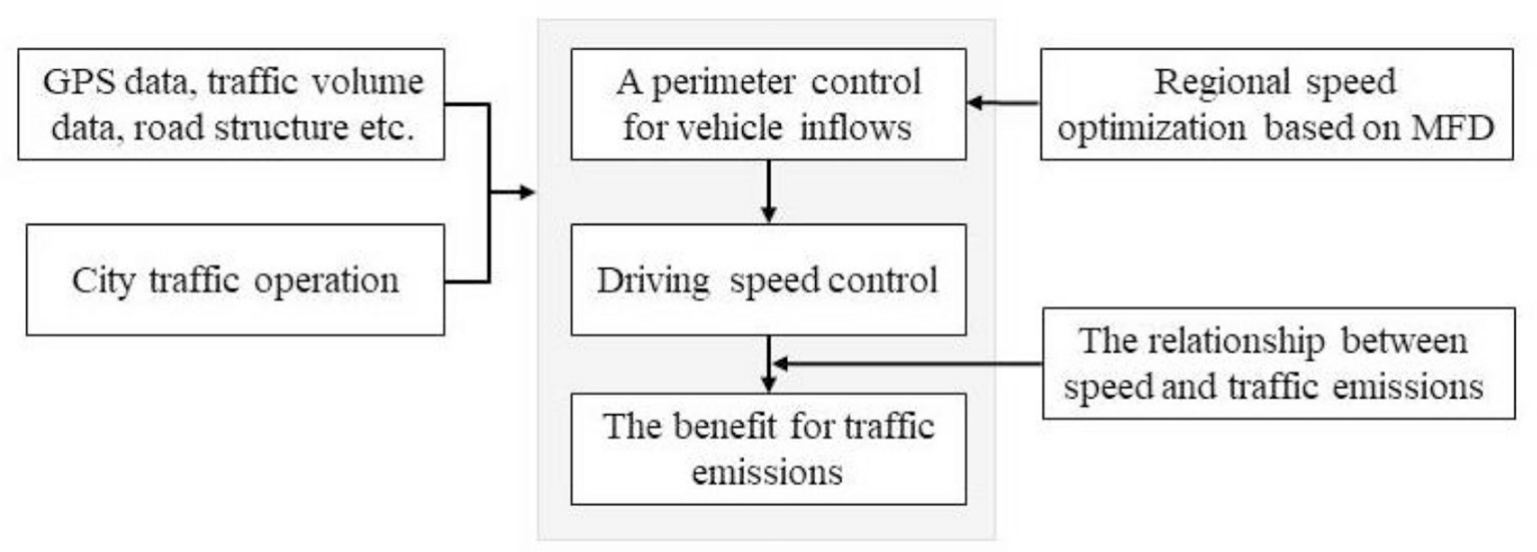
Overview of calculation process.

The following section describes the methodology and analysis process applied for this paper (Figure 1). By collecting traffic data such as GPS data of vehicles to obtain the actual city traffic operation state. The temporal and spatial characteristics of traffic operations in the urban road network can be found by analyzing historical data. A RSO strategy based on MFD which can control vehicles at optimized speeds was adopted to reduce transportation energy consumption and emissions across the road network. MFD is a model that shows the relationship between traffic flow and density in large urban networks. The formulations of road flow and density refer to Saffari et al. (34).

$q_{i}^{\prime }$(*t*) and $k_{i}^{\prime }$(*t*) are respectively partial flow and partial density of road *i*. *l*_*i*_ represent the length of road *i*. *n*_*i*_ denote the number of lanes of road *i*. *d*_*ip*_(*t*) and *t*_*ip*_(*t*) represent the travel distance and time spend by vehicle *p* on road *i*.


(1) $$q_{i}^{\prime }(t)=\frac{\sum_{p}d_{ip}(t)}{n_{i}l_{i}t}$$

(2) $$k_{i}^{\prime }(t)=\frac{\sum_{p}t_{ip}(t)}{n_{i}l_{i}t}$$

To obtain the traffic flow and density of the entire road network, it is indispensable to consider the penetration rate of GPS vehicle data. This study assumes the penetration rate ρ_*i*_ of vehicles with GPS. The flow *q*_*i*_(*t*) and density *k*_*i*_(*t*) of the complete road *i* can be calculated. The average traffic flow *Q*(*t*) and the average network density *K*(*t*) of the road network can be calculated by the following formulas.


(3) $$Q(t)=\frac{\sum_{p}q_{i}(t)l_{i}}{\sum l_{i}}$$

(4) $$K(t)=\frac{\sum_{p}k(t)l_{i}}{\sum l_{i}}$$

This paper expect to improve the speed of vehicles in the selected areas and ensure that the road capacity is fully utilized through a perimeter control strategy based on MFD.

Jiménez-Palacios (35) used vehicle specific power (VSP) as an intermediate parameter to establish a link between vehicle emissions and vehicle driving conditions, and the scientific and feasibility of this method were also demonstrated in the study. A very important property of this model is that it is measurable from the roadside. It can be used in scenarios where the number of vehicles is enormous, or the detailed information of all vehicles cannot be directly obtained. The simplified model is as follows.


(5) $$\begin{array}{r} VSP=\frac{1}{m}\left(A\cdot v+B\cdot v^{2}+C\cdot v^{3}\right)+\left(a+g\cdot sin\theta \right)\cdot v \end{array}$$

**v** and ***a*** represent the vehicle speed (*m*/*s*) and acceleration (*m*/*s*^2^), respectively. ***A***, ***B***, and ***C*** all represent vehicle or road-related coefficients, which can be measured experimentally, and the units are *kW*·*s*/*m*, kW·*s*^2^/*m*^2^, *kW*·*s*^3^/*m*^3^. ***m*** represents the mass of the vehicle of the specified model, the unit is ***t***. ***g*** is the acceleration of gravity. ***sin*θ** is the calculated value for the slope θ of the road. Through the parameters, we can find that the engine power of the vehicle is closely related to the speed and acceleration of the vehicle.

Trucks and container ships are only allowed to enter the Sixth Ring Road from 0:00 to 6:00 due to controls on the types of vehicles that travel in Beijing urban areas. To simplify the calculation, this study assumes that the vehicles driving in the urban area are all light-duty vehicles. The simplified formula applicable to light-duty vehicles in VSP methodology is used for the calculation [Equation (6)]. This formula is used by numerous studies (36–38).


(6) $$\begin{array}{r} VSP=\left(1.1a+9.81\cdot sin\theta +0.132\right)v+3.02\times 10^{-4}\cdot v^{3} \end{array}$$

Based on the above theory, this study proposes a road-vehicle specific power model. This model assumes that the road is the basic unit. In this way, the overall traffic operation state of the urban road network can be decomposed into the combination of roads in the road network. The first step is to obtain the historical traffic GPS data of the *R*_*i*_. The second step is to calculate the average velocity $A_{R_{i}}^{velocity}$ and acceleration $A_{R_{i}}^{acceleration}$ of the vehicle *A* on each road *R*_*i*_. With velocity *v* (unit of velocity *v* is *km*/*h*) as an interval, the vehicle driving data on the road *R*_*i*_ is divided into *V* (*V* = *velocity limit*/*v*) sets. The third step is to use the vehicle specific power formula to calculate the engine specific power value of each vehicle *A* traveling in this road *R*_*i*_. The vehicle specific power value reflects the traffic operation state on the road *R*_*i*_ at velocity *V*. Finally, divide the interval of the vehicle specific power value according to the step size *n*. The vehicle specific power distribution curve of the road *R*_*i*_ at vehicle velocity *v* can be obtained. The distribution curve can effectively reflect the urban traffic operation state on the completed road *R*_*i*_.


(7) $$VSP_{v,n}^{R_{i}}=\frac{\sum_{a=1}^{A}VSP_{v,n}^{R_{i},a}}{\sum_{v=1}^{speed\;limit}\sum_{n=1}^{N}\sum_{a=1}^{A}VSP_{v,n}^{R_{i},a}}$$

By establishing a corresponding specific power distribution curve for each road *R*_*i*_, different types of road segments I *R*_*i*_ can be effectively distinguished, and the relationship between urban built roads and traffic operations can be more accurately analyzed.


(8) $$\begin{array}{r} EF_{v}^{R_{i}}=\frac{\delta }{v}\sum_{j}ER_{j}^{R_{i}}\cdot D_{j}^{R_{i}} \end{array}$$

$EF_{v}^{R_{i}}$(*g*/*km*) is the traffic emission rate of road *R*_*i*_ at the average vehicle speed equal *v*. $ER_{j}^{R_{i}}$ (*g*/*km*) is the vehicle emission rate corresponding to the *j*th vehicle specific power interval in the specific power distribution curve of road *R*_*i*_. $D_{j}^{R_{i}}$ is the percentage of distribution of the *jth* specific power interval in the specific power distribution of road *R*_*i*_. δ is a constant with a value of 3600. Emission rates for different vehicle specific power have been well studied by Deng et al. (39). This paper uses the results of the Deng et al. (39) to calculate the road specific power emission rate $EF_{v}^{R_{i}}$. The average energy consumption rate of road *R*_*i*_ is estimated using the energy consumption rate $EC_{j}^{R_{i}}$ for each VSP mode *j*. The energy consumption rate corresponding to VSP has been fully studied in the Faria et al. research (2), and results were directly used for the calculation of energy consumption. $EC_{v}^{R_{i}}$ represents the road energy consumption rate of road *R*_*i*_ at speed *v*.


(9) $$\begin{array}{r} EC_{v}^{R_{i}}=\frac{\delta }{v}\sum_{j}EC_{j}^{R_{i}}\cdot D_{j}^{R_{i}} \end{array}$$

**Figure 2:**
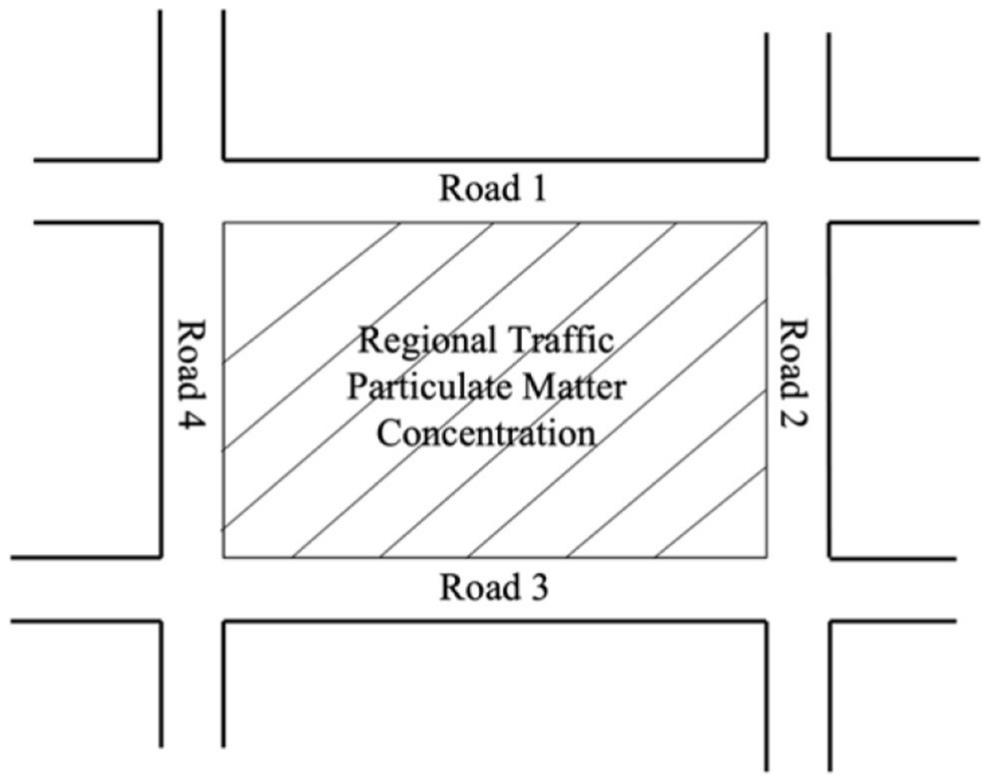
Regional emission concentration and road structure.

This study assumes that the traffic-PM2.5 pollution concentration in a built-up area is represented by the sum of the concentration produced by the four surrounding roads as shown in Figure 2. When joggers are running on the roadside of this area, this value is the PM2.5 value that affects the health of joggers.

### Data Collection

The vehicle GPS data time used in this study is from January 1 to 31 in 2021 of Beijing. The data content includes vehicle number, vehicle running state (driving or stopped), vehicle driving direction, vehicle speed, GPS coordinate point, time, and other information. The time interval for each GPS data is 3–5 s. In January 2021, the new crown pneumonia epidemic also occurred in China, so the traffic operation state reflected by the GPS data of vehicles may be different from the normal urban traffic operation state. The vehicle GPS data used in this study comes from our long-term collection and some data from the Beijing Municipal Transportation Administration.

**Figure 3:**
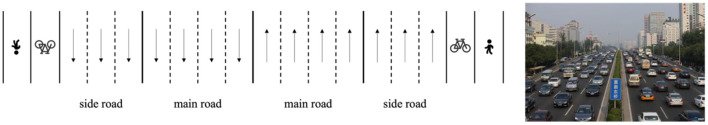
Structure of ring-road.

**Figure 4:**
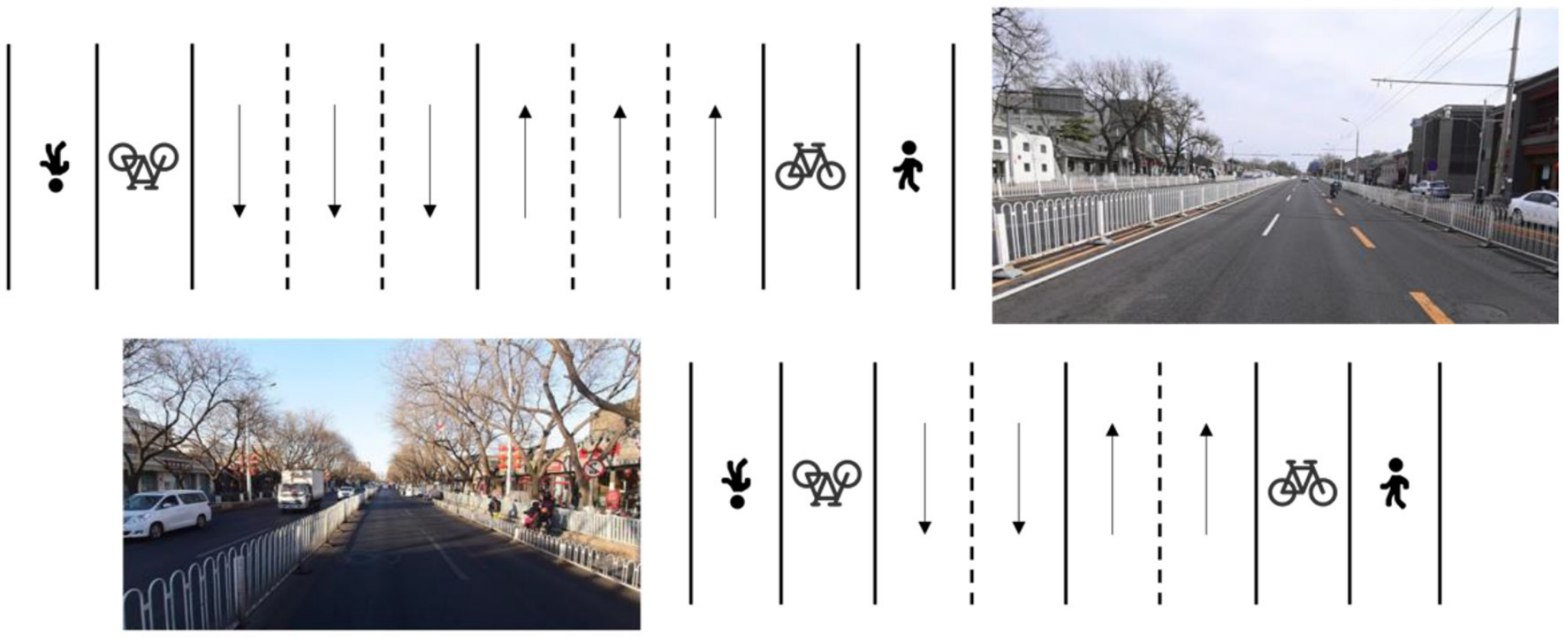
Structure of arterial road.

**Figure 5:**
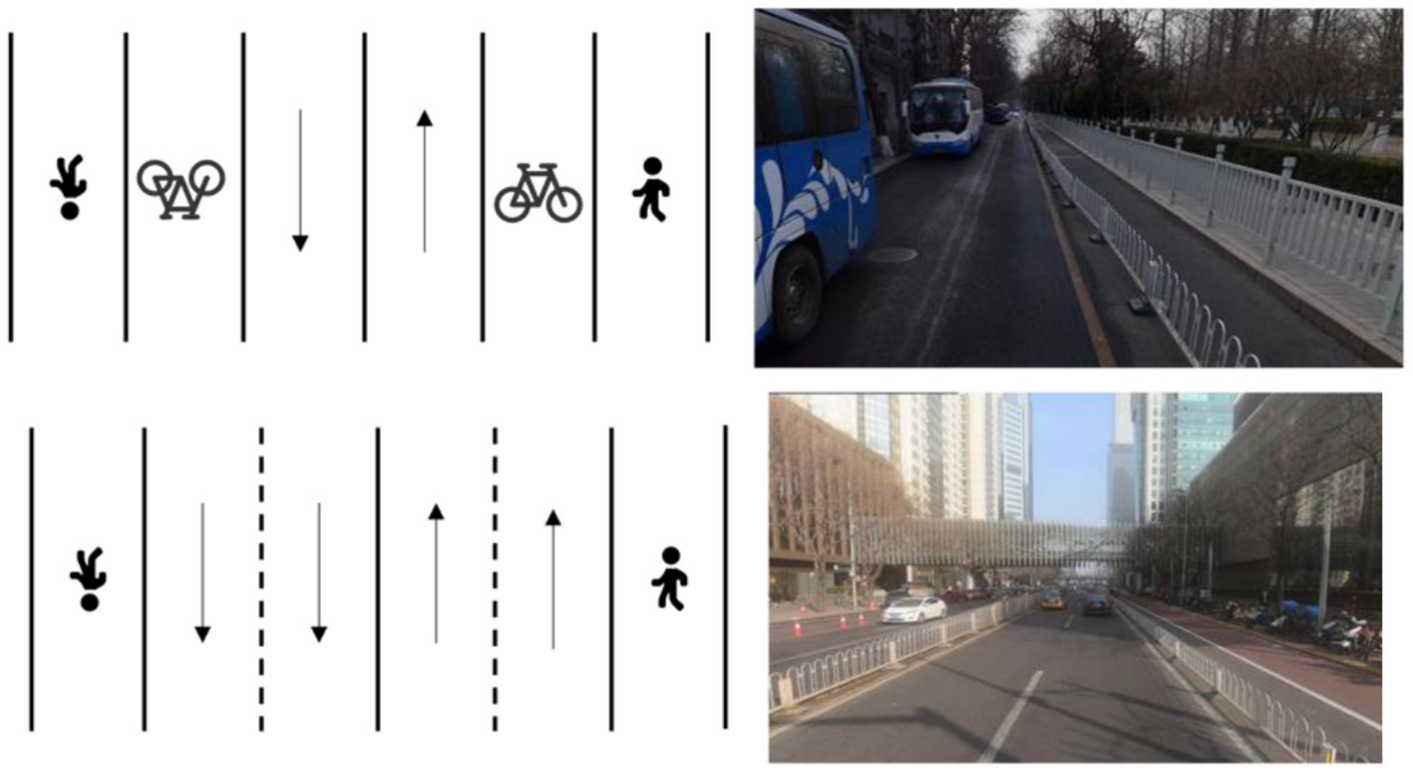
Structure of general road.

According to the existing built roads in Chinese cities, they can be divided into four levels: expressways, main roads, arterial roads, and general roads. Beijing was selected as our research object city. The Ring Road is one of the characteristics of urban built roads in Beijing (Figure 3). The Ring Road is divided into main road and auxiliary road. There are 7-8 lanes in a single direction, and it has great traffic capacity. The arterial road (Figure 4) has 2-3 lanes in a single direction, and there are dedicated bike lanes. A general road with only one lane in one direction or two lanes but no dedicated bike lanes (Figure 5). There are also differences in the maximum speed limits allowed and vehicle capacity on different classes of roads. The main reason for choosing Beijing as a case study is that Beijing has tens of thousands of online ride-hailing vehicles in operation in daily life. From the perspective of data acquisition, it is more convenient and can provide enough data to cover the entire road network of the city. Our method can be used in any other city if it is supported by sufficient vehicle data.

## Case Study and Result Analysis

### Case Description

**Figure 6:**
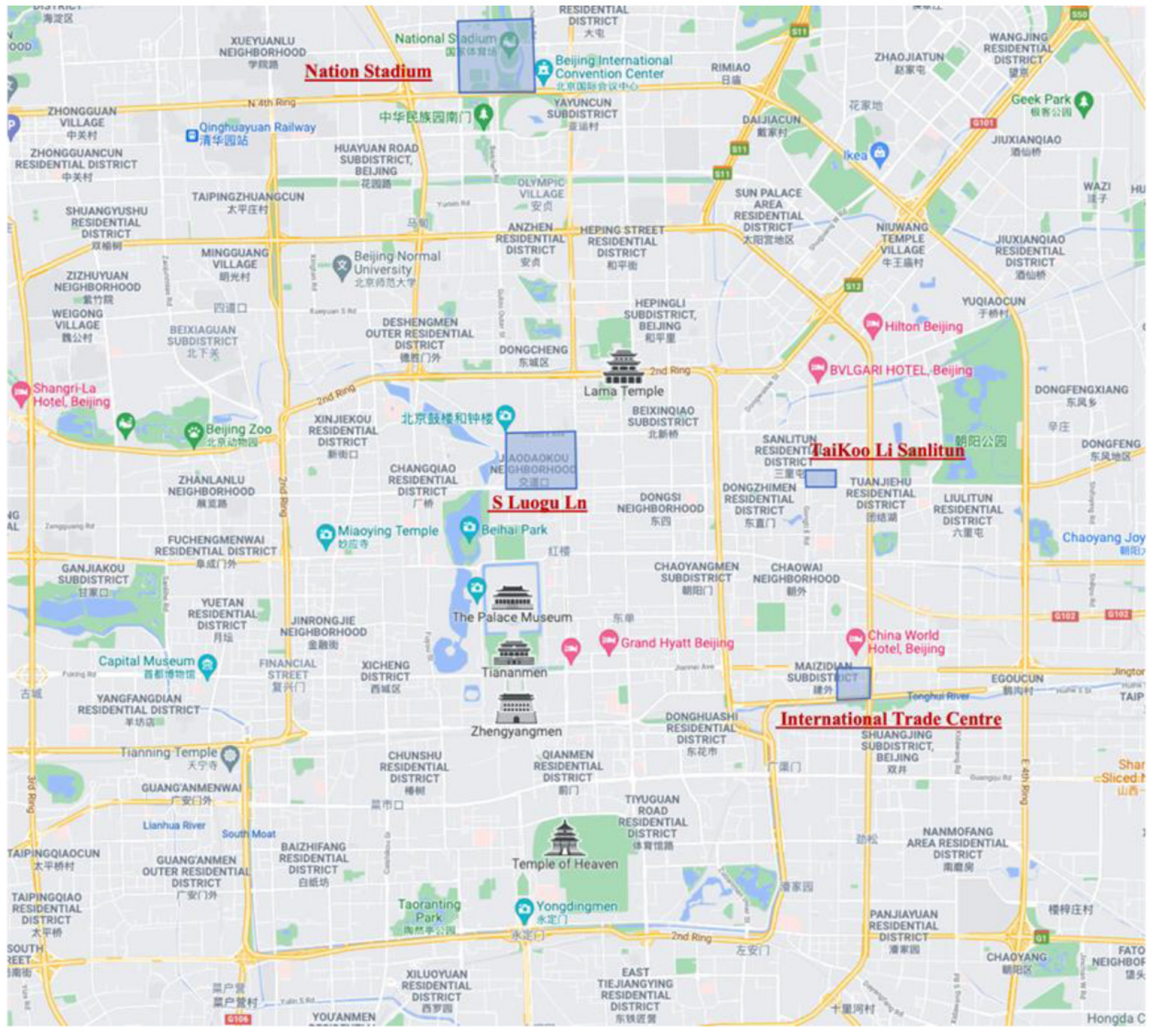
The location of the four areas and the names of the surrounding roads.

**Figure 7:**
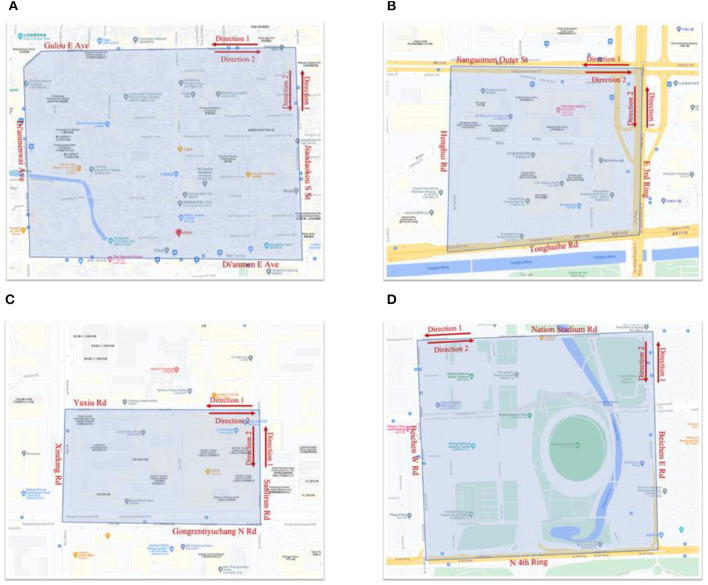
Four different area types and their surrounding roads. **(A)** Residential area-S Luogu Ln. **(B)** Sports area-nation stadium. **(C)** Shopping/entertainment area-sanlitun. **(D)** Business area-international trade centre.

As a super metropolis city with a population of more than 20 million, Beijing has a complex urban road structure and different urban built environments. Four typical urban areas as research objects to discuss the impact of traffic energy consumption and emissions on residents' health in different urban built environments. The four regions represent residential, commercial, entertainment, and park areas in the built environment of the city (Figures 6, 7). A brief introduction to the area is shown in Table 1.

**Table 1:** The name and structure of the built roads in the area.

**Area name**	**Road name**	**Number of lanes**	**Length of road (km)**
S Luogu Ln (Residential area)	Gulou E Ave	1	1
	Di'anmen E Ave	3	0.82
	Jiaodaokou S St	2	0.82
	Di'anmenwai Ave	1	1
Nation stadium (Sports area)	Nation Stadium Rd	2	1.2
	N 4th Ring	6	1.2
	Beichen E Rd	4	1.1
	Beichen W Rd	4	1.1
International trade centre (Business area)	Jianguomen Outer St	5	0.5
	Tonghuihe Rd	5	0.47
	E 3rd Ring	7	0.45
	Henghui Rd	2	0.5
Sanlitun (Shopping /entertainment area)	Yaxiu Rd	1	0.43
	Gongrentiyuchang N Rd	3	0.43
	Sanlirun Rd	1	0.24
	Xindong Rd	2	0.24

Gulou E Ave is one of the oldest neighborhoods in Beijing, with traditional Beijing Hutong-style buildings. The area is about 1,000 meters long in the east-west direction and 800 meters in the north-south direction. There are about 8,500 permanent residents in the community, most of whom are over 60 years old, and the number of tourists is about 20,000 every day. Most of the internal roads in this area are pedestrian roads, and the number of vehicles entering the interior is negligible. This area can be considered as a representative of the urban residential area.

The Guomao area is the CBD of Beijing, and the area selected in this study is a part of the CBD, with a length of about 500 m in both the east-west and north-south directions. There are over 40-story commercial office buildings in the area, such as Central World Trade Center, Jianwai SOHO and Beijing Yintai Center. The people in this area are mainly businesspeople who commute in the morning and evening.

TaiKoo Li Sanlitun is a prosperous entertainment area. The length of the area is about 420 m in the east-west direction and about 250 m in the north-south direction. There is a bar street in the area that is still very lively at night and traffic jams. There are also residential communities in this area, which is a mixed commercial and residential area.

The Olympic Sports Center is a large urban park. The area selected for this study is a small part of the Olympic Park, with a length of 1.2 km east-west and about 1.1 km north-south. The Water Cube and Bird's Nest are located here. The above-ground department in this area prohibits the passage of outside vehicles. It is one of the important places for urban residents to exercise and exercise.

### Median Speed Distribution of a Typical Road

**Figure 8:**
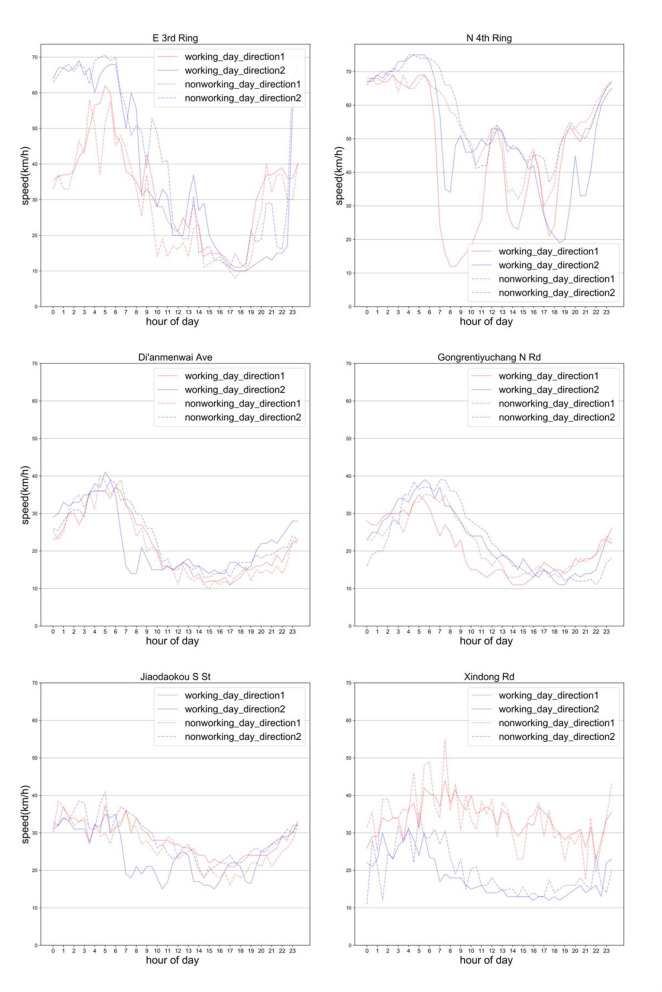
Median speed values for some typical roads in four regions.

By filtering the vehicle driving data of the selected road section, the median value of the vehicle speed on the road at half-hour intervals is obtained to represent the carried traffic operation state. Compared with the average speed, the median speed can better reflect the real situation of road driving. The result is shown in Figure 8. According to the traffic regulations formulated by Beijing, the speed limit on arterial and general roads is 60 *km*/*h*, the speed limit on Ring Roads is 80 *km*/*h*. Data that exceeds the maximum speed limit is culled from processing.

In Figure 8, the red and the blue curves represent the two driving directions of the same road, respectively. Solid and dashed lines are used to indicate the difference between working days and non-working days.

The surprising result is that the traffic operating state on the same road is also very different. Both the E 3rd Ring Road and N 4th Ring Road are part of Beijing Ring Road and have the same number of lanes, but have completely different median speed distributions during the day. On weekdays, the ring road, as the main traffic road in the city, starts to drop in speed from 6:00 in the morning, and the median speed drops rapidly between 7:00 and 9:00, which is also the early morning rush hour in Beijing. The minimum speed of the N 4th Ring appears in the east to west direction, about 12 *km*/*h*, which is lower than the 28–35 *km*/*h* of the E 3rd Ring. However, in the west to east direction of the Middle N 4th Ring, the minimum speed is about 36 *km*/*h* at about 8:30, and then quickly recovers to about 50 *km*/*h* within half an hour, which shows that the traffic in the west to east direction of the Middle N 4th Ring Congestion is more serious than west to east and lasts longer, and there is a tidal traffic phenomenon caused by commuting factors on the N 4th Ring during the morning rush hour. Through the median speed of the two driving directions during the evening rush hour (17:00 to 19:00) of the N 4th Ring Middle Road, it is found that during the evening rush hour, the congestion in the west-to-east direction is more serious than that in the east-to-west direction, which further confirms that this is due to the tidal phenomenon of traffic flow caused by commuting demand. This tidal phenomenon does not appear on the Middle E 3rd Ring. After 19:00 on the Middle E 3rd Ring, the speed of the south-to-north direction quickly rose to more than 30 *km*/*h*, while the middle of the north-to-south direction continued to maintain a low speed until 22:30. Middle E 3rd Ring has more demand to leave the core city at night, which is also in line with the characteristics of commuting.

On non-working days, the median speed of E 3rd Ring is almost the same as that on weekdays, which shows that E 3rd Ring is the main traffic artery in the city's road network. On the N 4th Ring, the median vehicle speed is above 40 *km*/*h* between 7:00 and 9:00 in the morning because there is no need for commuting on weekdays. This obvious tidal phenomenon on weekdays shows that this road section is not only the main arterial road in the city, but also a key road for urban commuting.

Di'anmen E Ave and Workers' Stadium North Road both have three lanes and have similar road structures. The median speed has a similar trend of change, but there are numerical differences. It can be seen from the Figure 8 that on weekdays, both roads start to drop in speed at around 6 o'clock and are in a state of low speed during the activity time. But on Di'anmen E Ave, after 10 o'clock, the median speed of the road is in a relatively balanced state, there is no strong fluctuation, only the east to west direction, there is a drop in speed during the evening rush hour, it is obvious that it is affected by the traffic flow in the evening rush hour, but the influence of the evening rush hour in another form direction is not obvious. On the North Road of the Workers' Stadium, the speed of the vehicle continued to drop from the beginning of people's activities, and maintained a low speed after 14:00, around 15 *km*/*h*, and continued to be below 20 *km*/*h* until 22:00, indicating that this road section at night, the speed of the car is slow, and it is more congested than Di'anmen E Ave, which is also closely related to Sanlitun being a commercial street. And from 19:00 to 22:00, there are differences in the driving directions of the two lanes, and the speed of the east-to-west direction is significantly lower than that of the west-to-east direction, indicating that there are more vehicles passing in this direction in the interval.

On non-working days, the speed of the east to west direction of Di'anmen E Ave decreases significantly between 11:00 and 18:00. This phenomenon occurs because although the Gulou E Ave area is an old Hutong block. The commercial pedestrian street attracts many tourists on weekends, so the increase in demand for rides in this direction leads to a decrease in the median speed.

Xindong Road is located on the west side of the Sanlitun area. It can be seen from the picture that there is a significant speed difference between the two driving directions of this road section, indicating that the traffic flow between the two driving directions of this road section is not balanced, and it is always in the south to north direction. The traffic flow is greater than the north-south direction.

Jianguomen Outer St is located on the extension line of the central axis of Chang'an Avenue and within the Second Ring Road, which belongs to the central area of Beijing. From the distribution of the median speed pair in the Figure 8, it can be found that the obvious tidal phenomenon between the two driving directions of this road section is even more serious than some sections of the N 4th Ring. As can be seen from the Figure 8, the speed of the east to west direction is significantly lower than that of the east to west direction, especially starting at 6:00 in the morning on weekdays, and starting at 8:30 in the morning on non-working days, within half an hour the vehicle speed will increase from 60 *km*/*h* rapidly dropped below 20 *km*/*h*, and the congestion formed rapidly within half an hour and continued until after 20 pm. In the west to east direction, although it also drops to 30 *km*/*h* from the morning peak period, the congestion is far less than that in the east to west direction. During the evening rush hour on weekdays, the east-west direction dropped rapidly from 30 *km*/*h* to 13 *km*/*h* and began to rapidly recover to 40 *km*/*h* after 19:00, indicating the impact of the increased traffic flow in the evening rush hour on the speed of the west-east direction. From the above results, we can conclude that for the Jianguomen Outer St section, whether it is the commuter flow on weekdays or the travel passenger flow on non-working days, the passenger flow into the city is the most important factor affecting the speed of this section, and because this section is in the second In the central area of the ring, the congestion began to gradually recover around 19:00 in the evening, and the recovery time was earlier than that of the third and fourth rings in the out-of-city direction (west to east).

### Emissions and Energy Consumption Characteristics of Typical Roads

Through the comparative analysis of the existing classification methods, this paper decides to use 1*kW*/*t* as the step size to compare the power for interval division. For light-duty vehicles in the urban road network, 99% of the vehicle specific power is distributed in the range of (-30, 30) kW/t, so the upper and lower limits of the vehicle specific power range (VSP bin) are set as−30kW, respectively /t and 30kW/t and divide the entire specific power range into 60. The specific power distribution curve of the road can reflect the traffic operating state of the road. The coordinate axes at the bottom are the traffic speed and specific power range of the vehicle, respectively.

**Figure 9:**
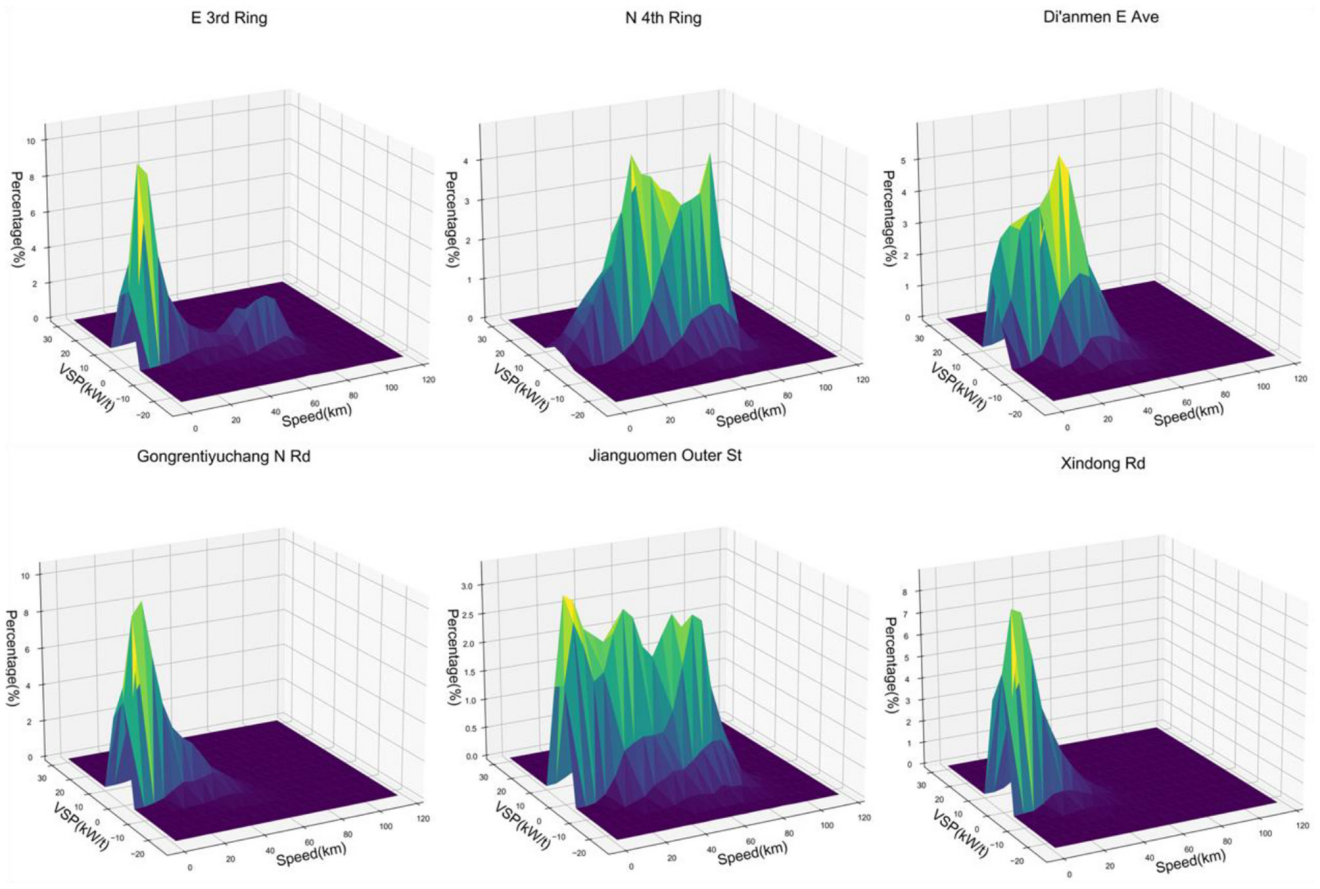
VSP distribution curve for some typical roads in four regions.

From the specific power curve of the above roads (Figure 9), it can be found that although the structures of the built roads may be similar, there are obvious differences in the specific power distribution curve on the roads due to different traffic operation states carry. For instance, the E 3rd Ring and the N 4th Ring Middle Road belong to the Ring Road. Most of the vehicles on the E 3rd Ring are in the low-speed operation area, and a considerable number of them are in the high-speed operation area of 60–80 *km*/*h*. However, on the section of the N 4th Ring Middle Road, there are fewer low-speed sections, and the distribution in the 20–80 *km*/*h* section is relatively even. This means that most of the time, the traffic operating state of the two roads is quite different. The specific power distribution curve of Workers' Stadium North Road and Xindong Road are similar, indicating that vehicles operating on the two road sections have similar traffic conditions and have similar traffic emission. Similar to the low-speed section of the Middle E 3rd Ring. The only difference is that the high-speed section is missing. This is due to the difference caused by the completion of the road structure. Since the road structure of the ring road is isolated (there is an isolation barrier from other roads), the maximum speed limit of the road is 80 *km*/*h*. On the other hand, roads such as Workers Stadium North Road and Xindong Road are mixed with pedestrians and non-motorized vehicles. To ensure safety, the speed cannot exceed 40 *km*/*h*.

Jianguomen Outer St is relatively evenly distributed in the range of 0–80 *km*/*h*. Though the low-speed range of 0–20 *km*/*h* accounts for the most, but each speed accounting for <3%. However, compared with the road section of the N 4th Ring, because the low-speed sections account for more, there are more inefficient driving states, so the emission intensity of the road section is also higher than that of the N 4th Ring.

**Figure 10:**
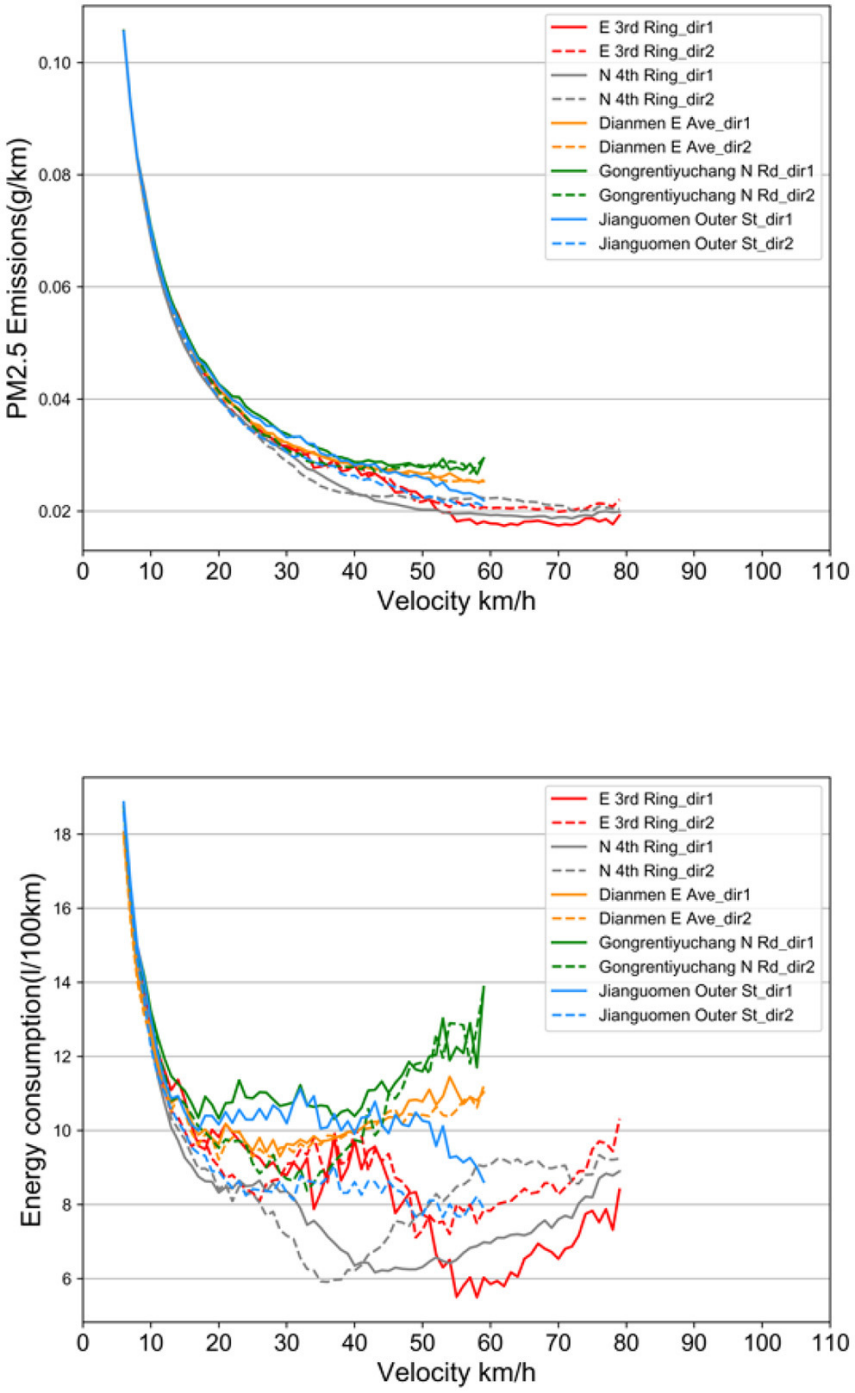
Relationship between PM2.5 emissions and energy consumption at different velocity.

Correlations between vehicle speed and energy consumption rate, vehicle speed and emission rate were obtained by using the VSP methodology shown in Figure 10. Among them, the VSP distributions of Xindong Rd and Gongrentiyuchang N Rd are similar, so Gongrentiyuchang N Rd is used to represent roads with similar VSP distributions.

Using the VSP methodology enabled correlating the average traffic emissions and fuel consumption of the road with each VSP mode and analyze speed bins at 1 *km*/*h* intervals. The maximum speed limit of the Ring Rd is 80 *km*/*h*, and the rest of the roads are limited to 60 *km*/*h*. Data on overspeed situations are excluded, and extreme low speed situations with vehicle speeds <5 *km*/*h* are also excluded. With the increase of driving speed from low speed to high speed, vehicle emissions and energy consumption have a trend of decreasing first and then increasing. The results of this study are corroborated by the conclusions of previous studies (2, 40). However, despite the same trends, the emissions and energy consumption of different roads differ significantly. This is due to the road structure of different roads, the importance of the road in the road network and the actual traffic operation state. Therefore, by using the method of this study, the relationship between speed and emissions and energy consumption can be established for each road in the road network using real data, which allows for large-scale driving monitoring activities at the urban road network level.

**Figure 11:**
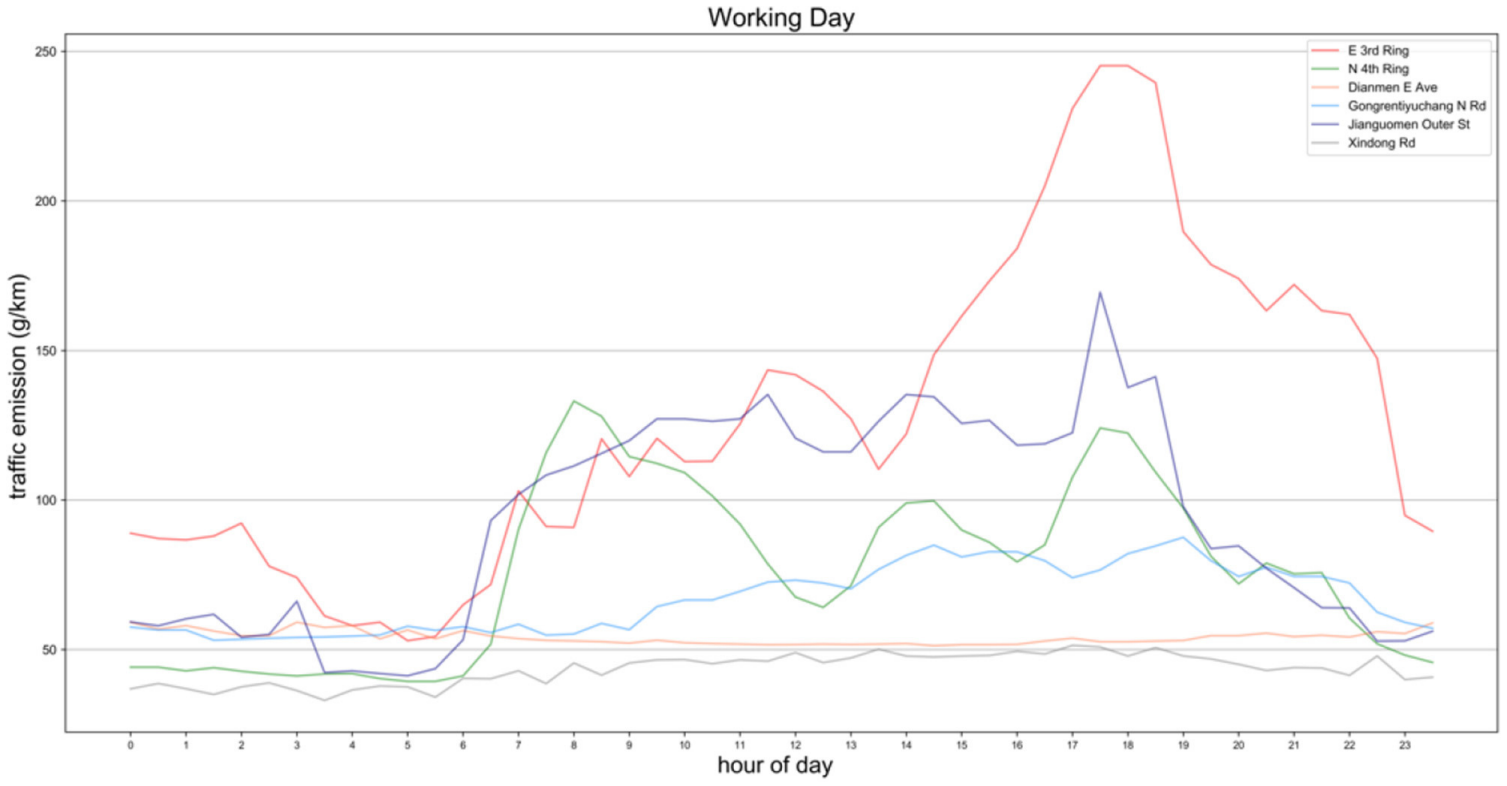
Working day traffic emissions from selected typical roads in four regions.

Figure 11 shows the emissions for selected road on weekdays, where traffic-related emissions from different driving directions of the road are superimposed to represent the overall emissions for that road segment. It can be seen from the Figure 11 that in the six roads, emissions began to rise at 6:00 in the morning. At this time, the morning commute began to appear, the speed of the road section began to drop, and the traffic flow increased significantly. Among them, the emissions of Middle E 3rd Ring, Middle N 4th Ring and Jianguomen Outer St have increased significantly, exceeding 100 *g*/*km*. This is also since the three roads have more lanes and undertake the main commuting function. From the emission curve of the N 4th Ring Middle Road, the emission during morning and evening peaks are more obvious. The maximum emission value of the morning peak is 162 *g*/*km*, which is slightly higher than that of the evening peak (148 *g*/*km*). Although there is an obvious commuter tidal traffic flow in this section on weekdays, However, in the morning and evening rush hour, the impact of the two driving directions of the road is relatively close. The highest emission value of the E 3rd Ring is close to 250 *g*/*km*, which occurs at 18:00 in the evening. The reason for this phenomenon is that the median speed of the East Third Ring Road is lower in the evening rush hour compared with the morning rush hour, and the congestion is more serious. PM2.5 emissions from traffic during evening rush hour are almost double those during morning rush hour. The emission from Jianguomen Outer St section began to rise rapidly from the time of residents' activity in the morning and remained between 120 and 135 *g*/*km* during the whole daytime. At 18:00 in the evening peak, it exceeded 150 *g*/*km* and began to decline continuously, indicating that this road section was active in the entire resident activity. It can be found from Figure 8 that the road also has obvious tidal phenomena, and in the period of daytime activity, the discharge from west to east is always higher than that from west to east. This result shows that the role of this road section in the entire traffic network is mainly to carry the traffic demand flowing to the city center.

The other three roads also have lower emissions than the above three roads due to the smaller number of one-way lanes. Gongrentiyuchang N Rd is close to the bustling shopping and entertainment district of Sanlitun, so the road speed is always low during the day. This resulted in emissions exceeding Xindong Road and Di'anmen E Ave.

### Application of Regional Speed Optimization Strategy

In this study, the MFD of four areas in Beijing is calculated by combining the vehicle GPS data, the fixed detectors data and video data on part of the networks. More details are given in Sarvi et al. (41). Utilizing the GPS data of actual driving vehicles is conducive to the establishment of a MFD that is closer to the actual road network traffic operation. Edie's generalized definitions (34) are used to calculate road flow and density.

Zhong et al. (42) investigated the dynamic system optimum (DSO) problem which simultaneous route and departure time assignments for a general traffic network partitioned into multiple regions. In this paper, MFD systems were constructed with the abscissa as the cumulative number of vehicles in the road network and the ordinate as the completed flow of the road network. The curve is represented by the third-order function of the cumulative number of vehicles in the road network, and its mathematical expression is:


(10) $$\begin{array}{r} G\left(n\left(t\right)\right)=\left(a\cdot n^{3}\left(t\right)+b\cdot n^{2}\left(t\right)+c\cdot n\left(t\right)+d\right)/3600 \end{array}$$

where *G*(*n*(*t*)) is the completed flow of the road network, and *n*(*t*) is the cumulative number of vehicles in the road network. *a, b, c, d* are the fitting parameters of the curve.

**Figure 12:**
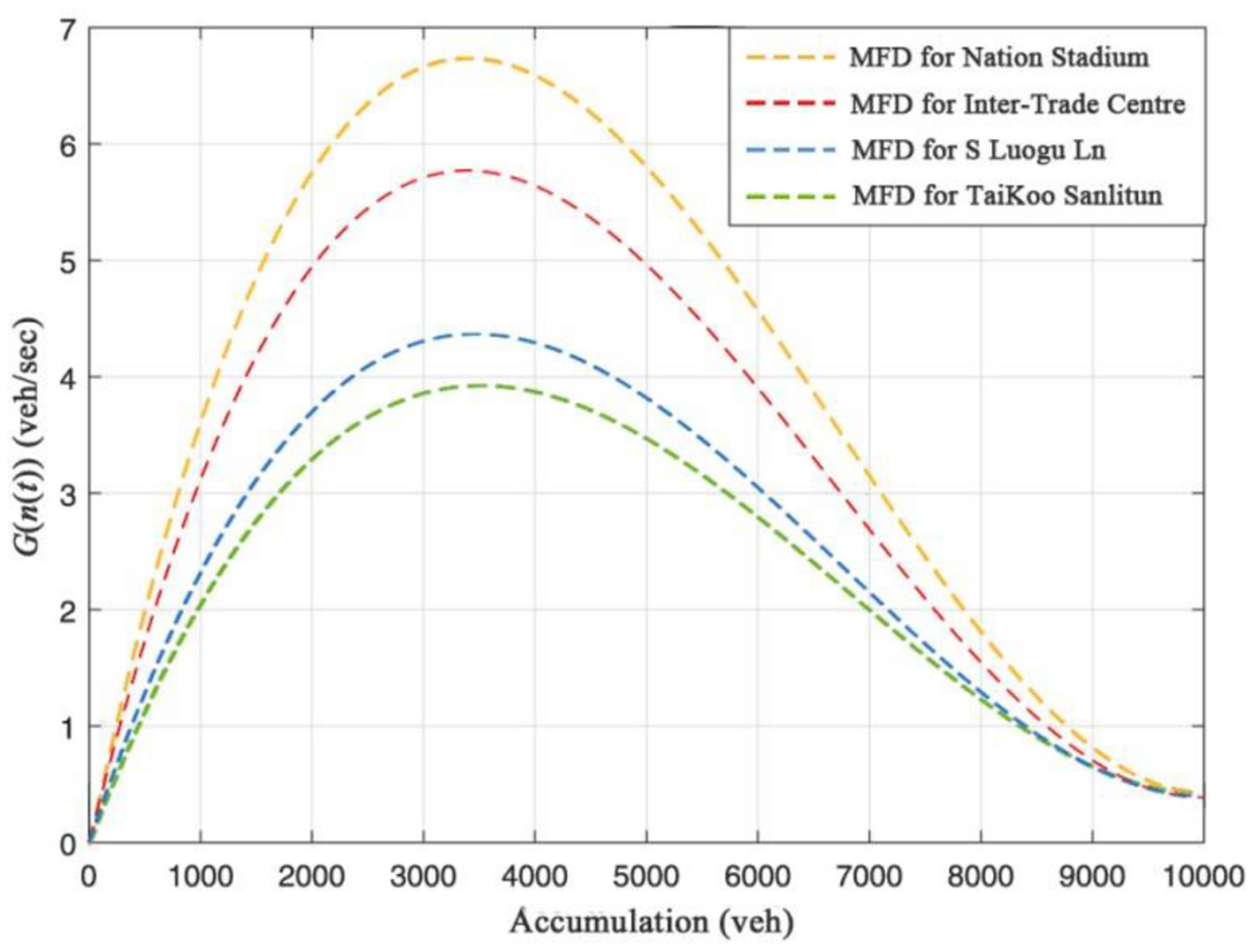
The estimated MFDs for the four regions.

**Figure 13:**
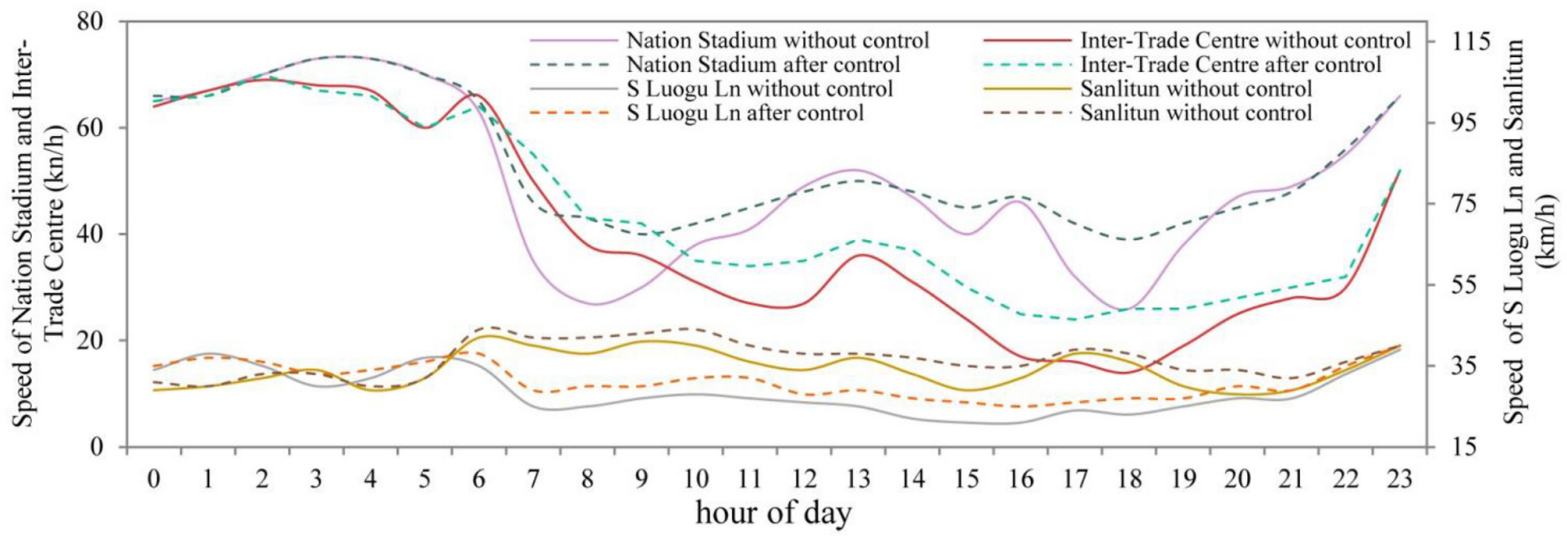
Median speed of the four regions before and after control.

In this study, four different types of areas were selected, and a simulation environment was built by VISSIM software to simulate the road traffic state of each area to calibrate the MFD system, and then obtain the best critical point for the cumulative number of vehicles in the road network. The estimated MFD systems for the four areas are shown in Figure 12. After that, a PID controller is used to control the cumulative number of vehicles in the road network around the optimal critical point. By controlling the number of vehicles entering each area, the speed of vehicles in the area is increased. The median speeds of the four areas before and after control are presented in Figure 13. It can be seen that the median speeds of the four areas are improved by the perimeter control strategy based on MFD. However, due to the constraints of the overall traffic flow in the road network (including the areas outside the control areas), the speed during peak hours cannot be increased to the level of off-peak hours. From the emissions and energy consumption results (Figure 10), low-speed operation caused by traffic congestion is a key factor affecting road energy consumption and emissions, especially during the morning and evening rush hours. By applying the RSO strategy to optimize the traffic operation status of the road network in different regions, the energy consumption of vehicles on the road network can be effectively reduced.

**Figure 14:**
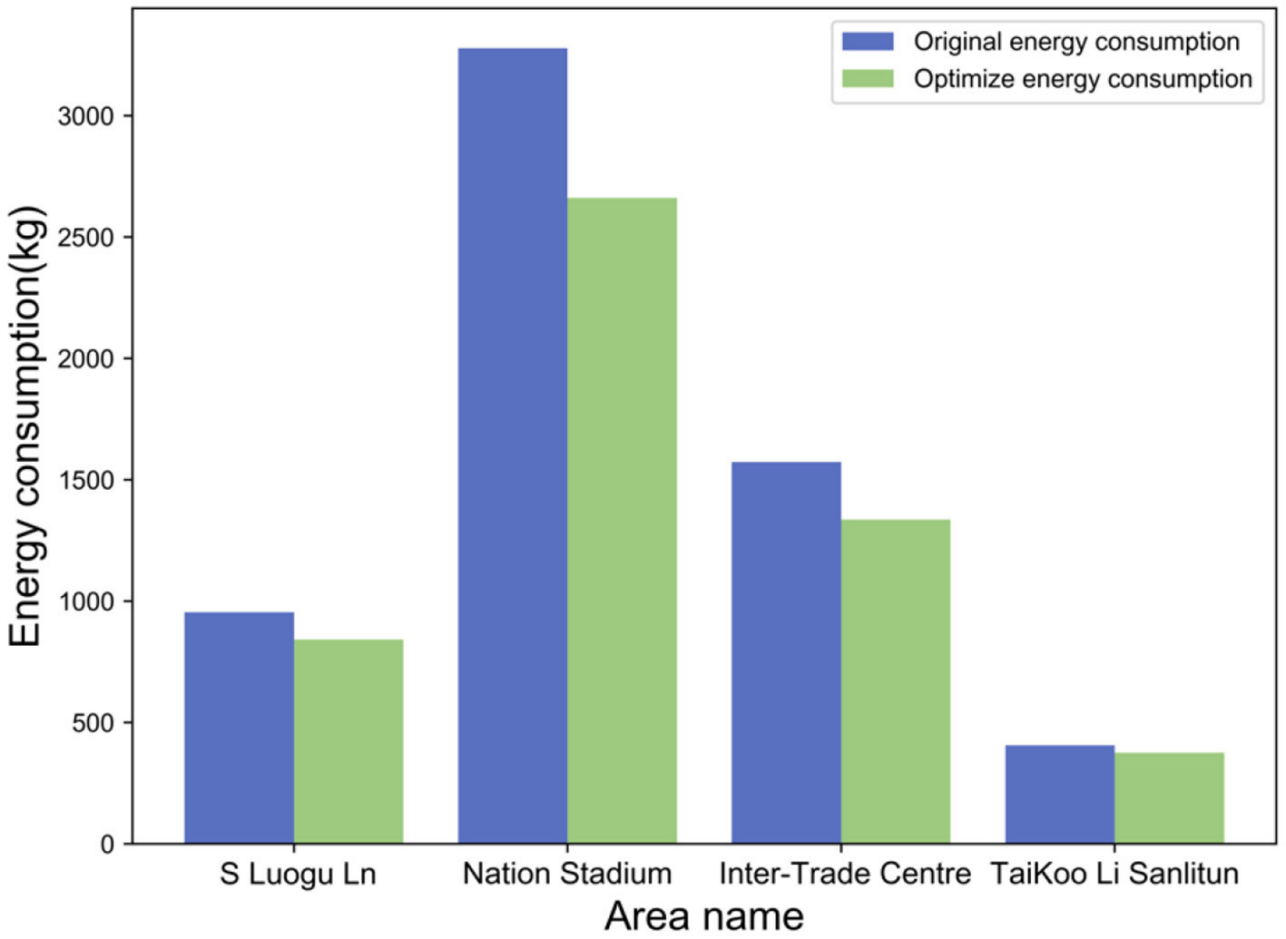
Four types of regional energy consumption results before and after RSO strategy optimization.

From the simulation results in Figure 14, it can be found that the energy consumption in the National Stadium area is highest (of the four areas) during the weekday evening peak hours. This is due to the traffic congestion caused by the excessive traffic demand on the N 4th Ring Road in the evening peak (17:00–19:00). After using the RSO strategy, vehicles flowing into the area from the surrounding area are controlled. The traffic flow in the area decreased and the driving speed increased. The overall energy consumption of road network traffic in the region fell by about 18.8%, the largest drop among the four regions. The second is the international trade area, where the overall energy consumption has dropped by about 15%. S Luogu Ln and Sanlitun area cities have few lanes in the built road network, the RSO strategy has limited improvement in the vehicle traffic capacity of the road network. The reduction in energy consumption decreased by about 11.7 and 7.5%, respectively. In the urban road network construction structure, the area with the greater traffic capacity, the more obvious the energy consumption reduction effect after using the RSO strategy.

**Figure 15:**
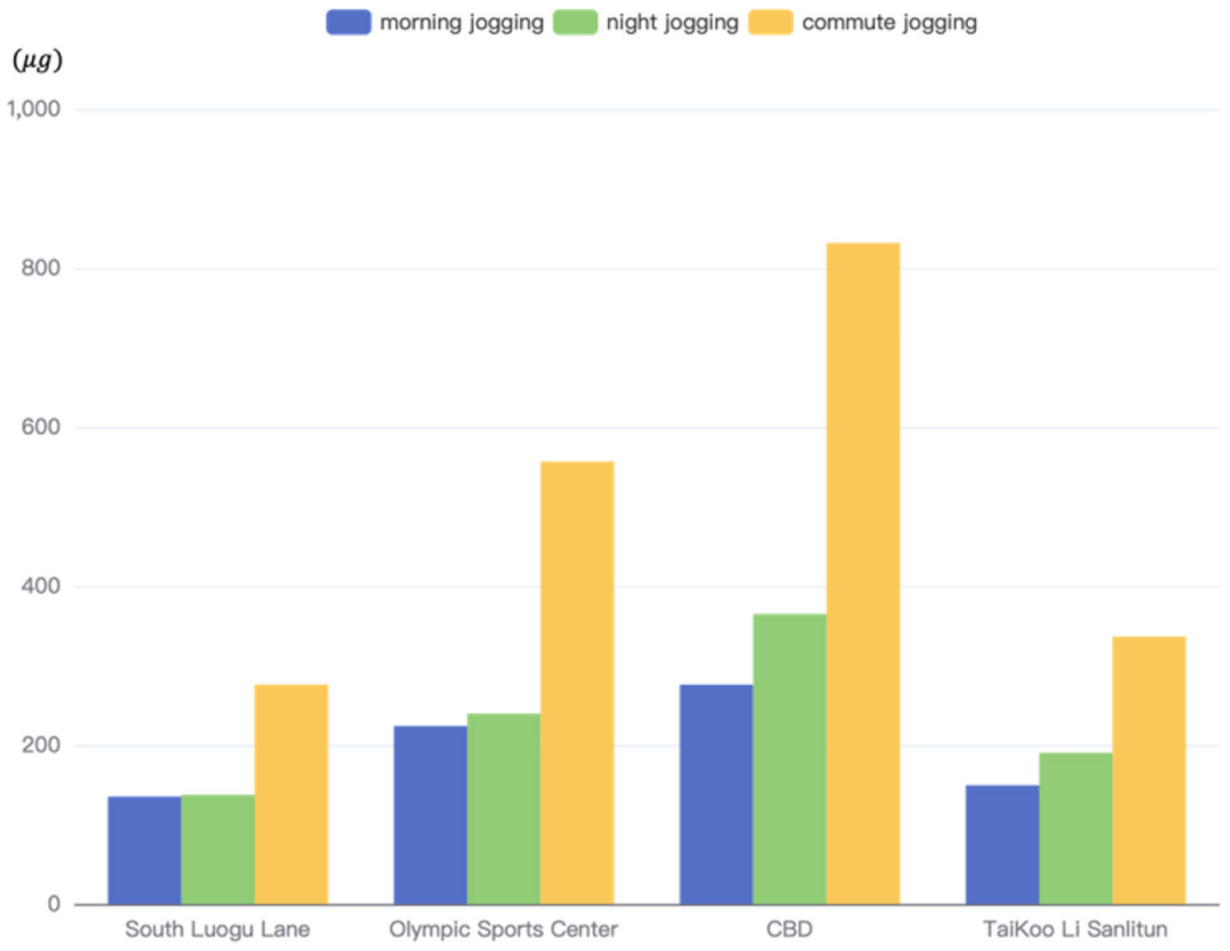
PM2.5 inhalation caused by jogging on some typical roads in four regions.

Roadside jogging is one of the typical behaviors of roadside pedestrians (43, 44), and getting too close to the road is more susceptible to traffic-related PM2.5 emissions. To explore the impact of traffic emissions on the fitness activities of residents on the PM2.5, this paper calculated the PM2.5 inhalation during jogging activities in different functional areas at different times of the working day. We assume that there are three types of runners: morning runners, night runners, and commuter runners. Different types of running times were obtained by reading the literature (45, 46). The morning jogging is from 6:00 to 7:00 in the morning, and the night jogging is from 20:00 to 21:00. For those who commute by running, it is assumed that the commute time is 1 h in the morning and evening, between 7:00 and 8:00 in the morning, and between 18:00 and 19:00 in the evening (47). The concentrations of morning and evening commuter runs were then averaged and compared to those who only ran in the morning or evening. Running and inhalation studies were used to estimate inhalation in jogging behavior (48). The average speed during jogging behavior was 8 *km*/*h* and the inspiratory rate was 1.62 *m*^3^/*h*. The calculation results are shown in Figure 15. Through calculation, it can be found that the changes in traffic emissions suffered by physical exercise in different regions and time periods are different.

When runners jogged around four different types of zones, the cumulative PM2.5 concentrations they were exposed to within 1 h were significantly different. The International Trade Centre area is a CBD with busy business activities. From the previous median speed results, it can be found that because the selected area is close to the Middle E 3rd Ring, the traffic flow in this area is large, and the passing vehicles are huge. So the value of PM2.5 concentration is also the highest among the four areas. The PM2.5 concentration generated by traffic during night jogging activities in this area is 1.32 times that of morning jogging activities. It is 2.65 times that of night running in the S Luogu Ln area (residential living area).

In the commuting and running behavior in the International Trade Centre area, the cumulative amount of PM2.5 inhaled by runners exceeds 800 μ*g*. Combined with previous research, running in this polluted environment is more harmful to health than exercise. The shopping malls, gourmet shops and bars in the area make the Sanlitun area more active at night than during the daytime. Therefore, the PM2.5 inhalation of night jogging (190 μ*g*) in this area is higher than that of morning jogging (150 μ*g*). In the S Luogu Ln area, whether it is morning or night jogging, the PM2.5 inhalation was almost the same, 135 μ*g* and 137 μ*g*, respectively. Due to the small traffic capacity of the built roads in the residential area, the emission from traffic is small, which is the main factor for the small fluctuation of the PM2.5 concentration value around the residential area. To sum up, jogging exercise is best done around residential areas without major urban roads. Other exercise methods such as cycling or walking have breathing rates of 0.63 and 0.70, respectively, which are much lower than running (49). Therefore, engaging in lower-breathing exercise activities can help your health when exposed to polluted outdoor environments. For runners, try to run in residential areas and avoid jogging activities in commercial areas and main roads. And take precautions to reduce PM2.5 inhalation while jogging. Of course, the most ideal sports venue is the professional track and field ground.

## Conclusions

Although the built roads have the same number of lanes, there are obvious differences in the traffic operating state on the road because they are in different areas of the urban road network. One of the reasons is the tidal phenomenon of traffic flow caused by different commuting needs. By using the emission and energy consumption model proposed in this study, the energy consumption and emission results of roads in four different regions are calculated. Compared with the maximum hourly emission of PM2.5 generated by traffic on weekdays, the East Third Ring Road is 1.66 times that of the N 4th Ring Road. Therefore, the emissions and ambient PM2.5 concentrations of roads that play an important role in the road network deserve further study. We will study this aspect in depth in the future. In addition, this study found that the PM2.5 inhalation of commuter running is about 2-3 times that of morning or night runners alone. Traffic emissions and energy consumption can be effectively reduced by increasing vehicle speed during evening rush hour. By simulating the road network in four regions, energy consumption can be reduced by up to 18.8% Through the method proposed in this study, the RSO strategy can be extended to the entire road network, which can effectively reduce the energy consumption and emissions of urban traffic.

According to the study of Lu et al. (14), if jogging on the roadside for a long time, even in a residential area, the PM2.5 generated by traffic can increase the risk of commuters jogging suffering from respiratory related diseases. About 10.3% higher risk than just jogging in the morning or evening. Commuter runners should take some precautions to reduce the impact of PM2.5 emissions from traffic. We strongly recommend that runners avoid arterial urban roads as much as possible. Try to avoid the rush hour of traffic in the choice of time. It is best to run in a dedicated stadium.

This paper may provide valuable information for the study of the relationship between the urban built environment and the health of urban residents.

Our findings may provide valuable information for the study of the relationship between the urban built environment and the health of urban residents. It can also play a role in helping to formulate sustainable air pollution control policies to protect the health of future urban residents. Nevertheless, this study still has some limitations that need to be further expanded in the future. In the emission research at the level of urban road network, a larger amount of data and more detailed vehicle driving data are helpful to describe the traffic operation state and improve the accuracy of emission calculation more accurately. More data needs to be collected for analysis and research. Sophisticated wearable detection equipment is also helpful to analyze the impact of traffic emissions on particulate matter inhaled by roadside pedestrians. These issues will be further improved in future research.

## Data Availability Statement

The raw data supporting the conclusions of this article will be made available by the authors, without undue reservation.

## Author Contributions

WW: modified the models and solved many problems to improve the manuscript substantively. ZW: conceptualization and writing—original draft. BY: supervision. YX, BY, and ZW: methodology. YX: programming. KY and GW: investigation and data collection. All authors contributed to the article and approved the submitted version.

## Funding

This research was supported by National Natural Science Foundation of China (U1811463).

## Conflict of Interest

ZW was employed by CIECC Overseas Consulting Co., Ltd. YX was employed by China Railway Construction Investment Group Co., Ltd. The remaining authors declare that the research was conducted in the absence of any commercial or financial relationships that could be construed as a potential conflict of interest.

## Publisher's Note

All claims expressed in this article are solely those of the authors and do not necessarily represent those of their affiliated organizations, or those of the publisher, the editors and the reviewers. Any product that may be evaluated in this article, or claim that may be made by its manufacturer, is not guaranteed or endorsed by the publisher.

## References

[1] Fotouhi A Yusof R Rahmani R Mekhilef S Shateri N. A review on the applications of driving data and traffic information for vehiclesenergy conservation. Renew Sustain Energy Rev. (2014) 37:822–33. 10.1016/j.rser.2014.05.077

[2] Faria M Rolim C Duarte G Farias T Baptista P. Assessing energy consumption impacts of traffic shifts based on real-world driving data. Transport Res D Transport Environ. (2018) 62:489–507. 10.1016/j.trd.2018.03.008

[3] Li Y Lv C Yang N Liu H Liu Z. A study of high temporal-spatial resolution greenhouse gas emissions inventory for on-road vehicles based on traf fi c speed- fl ow model : a case of Beijing. J Clean Prod. (2020) 277:122419. 10.1016/j.jclepro.2020.122419

[4] Wang H Colvile RN Pain C Aristodemou E Apsimon HM. Understanding peak pedestrian exposures due to traffic emissions within the urban environment. Transport Res D Transport Environ. (2011) 16:392–401. 10.1016/j.trd.2011.03.002

[5] Yu Z Li W Liu Y Zeng X Zhao Y Chen K. Quantification and management of urban traffic emissions based on individual vehicle data. J Clean Prod. (2021). 328:129386. 10.1016/j.jclepro.2021.129386

[6] Salma I Füri P Németh Z Balásházy I Hofmann W Farkas Á. Lung burden and deposition distribution of inhaled atmospheric urban ultrafine particles as the first step in their health risk assessment. Atmos Environ. (2015) 104:39–49. 10.1016/j.atmosenv.2014.12.060

[7] Szyszkowicz M Kousha T Castner J Dales R. Air pollution and emergency department visits for respiratory diseases: a multi-city case crossover study. Environ Res. (2018) 163:263–9. 10.1016/j.envres.2018.01.043 29459308

[8] Zauli-Sajani S Rovelli S Trentini A Bacco D Marchesi S Scotto F. Higher health effects of ambient particles during the warm season: the role of infiltration factors. Sci Total Environ. (2018) 627:67–77. 10.1016/j.scitotenv.2018.01.217 29426191

[9] Chen L Zhang Y Zhang W Chen G Lu P Guo Y. Short-term effect of PM1 on hospital admission for ischemic stroke: a multi-city case-crossover study in China. Environ Pollut. (2020) 260:113776. 10.1016/j.envpol.2019.113776 31962264

[10] Liu C Chen R Sera F Vicedo-Cabrera AM Guo Y Tong S. Ambient particulate air pollution and daily mortality in 652 cities. N Eng J Med. (2019) 381:705–15. 10.1056/NEJMoa1817364 31433918 PMC7891185

[11] Lin C Hu D Jia X Chen J Deng F Guo X. The relationship between personal exposure and ambient PM 2. 5 and black carbon in Beijing. Sci Total Environ. (2020) 737:139801. 10.1016/j.scitotenv.2020.139801 32783824

[12] Chen D Mayvaneh F Baaghideh M Entezari A Ho HC Xiang Q. Utilizing daily excessive concentration hours to estimate cardiovascular mortality and years of life lost attributable to fine particulate matter in Tehran, Iran. Sci Total Environ. (2020) 703:134909. 10.1016/j.scitotenv.2019.134909 31757557

[13] Yang BY Guo Y Morawska L Bloom MS Markevych I Heinrich J. Ambient PM1 air pollution and cardiovascular disease prevalence: insights from the 33 communities Chinese health study. Environ Int. (2019) 123:310–7. 10.1016/j.envint.2018.12.012 30557810

[14] Lu F Xu D Cheng Y Dong S Guo C Jiang X. Systematic review and meta-analysis of the adverse health effects of ambient PM2.5 and PM10 pollution in the Chinese population. Environ Res. (2015) 136:196–204. 10.1016/j.envres.2014.06.029 25460637

[15] Karusisi N Bean K Oppert J Pannier B Chaix B. Multiple dimensions of residential environments, neighborhood experiences, and jogging behavior in the RECORD Study. Prev Med. (2012) 55:50–5. 10.1016/j.ypmed.2012.04.018 22564774

[16] Yang L Liu J Liang Y Lu Y Yang H. Spatially varying effects of street greenery on walking time of older adults. ISPRS Int J Geo-Inform. (2021) 10:596. 10.3390/ijgi10090596

[17] Yang L Ao Y Ke J Lu Y Liang Y. To walk or not to walk? examining non-linear effects of streetscape greenery on walking propensity of older adults. J Transp Geogr. (2021) 94:103099. 10.1016/j.jtrangeo.2021.103099

[18] Laña I Del Ser J Padró A Vélez M Casanova-Mateo C. The role of local urban traffic and meteorological conditions in air pollution: a data-based case study in Madrid, Spain. Atmos Environ. (2016) 145:424–38. 10.1016/j.atmosenv.2016.09.052

[19] Weichenthal S Van Ryswyk K Goldstein A Shekarrizfard M Hatzopoulou M. Characterizing the spatial distribution of ambient ultrafine particles in Toronto, Canada: a land use regression model. Environ Pollut. (2016) 208:241–8. 10.1016/j.envpol.2015.04.011 25935348

[20] Deng Q Deng L Miao Y Guo X Li Y. Particle deposition in the human lung: health implications of particulate matter from different sources. Environ Res. (2019) 169:237–45. 10.1016/j.envres.2018.11.014 30476747

[21] Ling S Ma S Jia N. Sustainable urban transportation development in China: a behavioral perspective. Front Eng Manag. (2022) 9:16–30. 10.1007/s42524-021-0162-4

[22] Ao Y Zhang Y Wang Y Chen Y Yang L. Influences of rural built environment on travel mode choice of rural residents: the case of rural Sichuan. J Transp Geogr. (2020) 85:102708. 10.1016/j.jtrangeo.2020.102708

[23] Bereitschaft B. Pedestrian exposure to near-roadway PM 2. 5 in mixed-use urban corridors : a case study of Omaha, Nebraska. Sustain Cities Soc. (2015). 15:64–74. 10.1016/j.scs.2014.12.001

[24] Jia S. Economic, environmental, social, and health benefits of urban traffic emission reduction management strategies : case study of Beijing, China. Sustain Cities Soc. (2021) 67:102737. 10.1016/j.scs.2021.102737

[25] Wu Z He Q Chen Q Xue H Li S. A topical network based analysis and visualization of global research trends on green building from 1990 to 2020. J Clean Prod. (2021) 320:128818. 10.1016/j.jclepro.2021.128818

[26] Chong HS Kwon S Lim Y Lee J. Real-world fuel consumption, gaseous pollutants, and CO2 emission of light-duty diesel vehicles. Sustain Cities Soc. (2020) 53:101925. 10.1016/j.scs.2019.10192522893964

[27] Zhang S Wu Y Liu H Huang R Un P Zhou Y. Real-world fuel consumption and CO2 (carbon dioxide) emissions by driving conditions for light-duty passenger vehicles in China. Energy. (2014) 69:247–57. 10.1016/j.energy.2014.02.103

[28] Brundell-Freij K Ericsson E. Influence of street characteristics, driver category and car performance on urban driving patterns. Transport Res D Transport Environ. (2005) 10:213–29. 10.1016/j.trd.2005.01.001

[29] Chong HS Park Y Kwon S Hong Y. Analysis of real driving gaseous emissions from light-duty diesel vehicles. Transport Res D Transport Environ. (2018) 65:485–99. 10.1016/j.trd.2018.09.015

[30] De Vlieger I De Keukeleere D Kretzschmar JG. Environmental effects of driving behaviour and congestion related to passenger cars. Atmos Environ. (2000) 34:4649–55. 10.1016/S1352-2310(00)00217-X

[31] Fontaras G Franco V Dilara P Martini G Manfredi U. Development and review of Euro 5 passenger car emission factors based on experimental results over various driving cycles. Sci Total Environ. (2014) 468–9:1034–42. 10.1016/j.scitotenv.2013.09.043 24095966

[32] Li B Lei X-n Xiu G-l Gao C-y Gao S Qian N. Personal exposure to black carbon during commuting in peak and off-peak hours in Shanghai. Sci Total Environ. (2015). 524–5:237–45. 10.1016/j.scitotenv.2015.03.088 25909267

[33] Marmett B Pires Dorneles G Böek Carvalho R Peres A Roosevelt Torres Romão P Barcos Nunes R. Air pollution concentration and period of the day modulates inhalation of PM2.5 during moderate- and high-intensity interval exercise. Environ Res. (2021) 194:110528. 10.1016/j.envres.2020.110528 33248052

[34] Saffari E Yildirimoglu M Hickman M. Data fusion for estimating Macroscopic Fundamental Diagram in large-scale urban networks. Transp Res Part C Emerg Technol. (2022) 137:103555. 10.1016/j.trc.2022.103555

[35] Jiménez-Palacios JL. Understanding and Quantifying Motor Vehicle Emissions With Vehicle Specific Power and TILDAS Remote Sensing. Massachusetts Institute of Technology, Cambridge (1999). p. 361.

[36] Duarte GO Gonçalves GA Farias TL. A methodology to estimate real-world vehicle fuel use and emissions based on certification cycle data. Proc Soc Behav Sci. (2014) 111:702–10. 10.1016/j.sbspro.2014.01.104

[37] He K Huo H Zhang Q He D An F Wang M. Oil consumption and CO2 emissions in China's road transport: current status, future trends, and policy implications. Energy Policy. (2005) 33:1499–507. 10.1016/j.enpol.2004.01.007

[38] Huo H Yao Z He K Yu X. Fuel consumption rates of passenger cars in China: Labels versus real-world. Energy Policy. (2011) 39:7130–5. 10.1016/j.enpol.2011.08.031

[39] Deng F Lv Z Qi L Wang X Shi M Liu H. A big data approach to improving the vehicle emission inventory in China. Nat Commun. (2020) 11:1–12. 10.1038/s41467-020-16579-w 32493934 PMC7271216

[40] Ntziachristos L Gkatzoflias D Kouridis C Mellios G Geivanidis S Samaras Z. Copert 4. Mechanical Engineering (2008).

[41] Sarvi M Horiguchi R Kuwahara M Shimizu Y Sato A Sugisaki Y. A methodology to identify traffic condition using intelligent probe vehicles. In: Proceedings of 10th ITS World Congress, Madrid (2003). p. 17–21.

[42] Zhong R Xiong J Huang Y Sumalee A Chow AHF Pan T. Dynamic system optimum analysis of multi-region macroscopic fundamental diagram systems with state-dependent time-varying delays. IEEE Trans Intell Transp Syst. (2020) 21:4000–16. 10.1109/TITS.2020.2994347

[43] Liu Y Hu J Yang W Luo C. Effects of urban park environment on recreational jogging activity based on trajectory data : a case of Chongqing, China. Urban For Urban Green. (2022) 67:127443. 10.1016/j.ufug.2021.127443

[44] Qviström M Fridell L Kärrholm M. Differentiating the time-geography of recreational running. Mobilities. (2020) 15:575–87. 10.1080/17450101.2020.1762462

[45] Peng L Shen Y Gao W Zhou J Pan L Kan H. Personal exposure to PM2.5 in five commuting modes under hazy and non-hazy conditions. Environ Pollut. (2021) 289:117823. 10.1016/j.envpol.2021.117823 34325093

[46] Zhang M He S Zhao P. Revisiting inequalities in the commuting burden: Institutional constraints and job-housing relationships in Beijing. J Transp Geogr. (2018) 71:58–71. 10.1016/j.jtrangeo.2018.06.024

[47] Yang L Ding C Ju Y Yu B. Driving as a commuting travel mode choice of car owners in urban China: roles of the built environment. Cities. (2021) 112:103114. 10.1016/j.cities.2021.103114

[48] Liu B He MM Wu C Li J Li Y Lau NT. Potential exposure to fine particulate matter (PM2.5) and black carbon on jogging trails in Macau. Atmos Environ. (2019) 198:23–33. 10.1016/j.atmosenv.2018.10.024

[49] Li Y Henze DK Jack D Henderson BH Kinney PL. Assessing public health burden associated with exposure to ambient black carbon in the United States. Sci Total Environ. (2016) 539:515–25. 10.1016/j.scitotenv.2015.08.129 26383853 PMC4761114

